# Climate Extreme Effects on the Chemical Composition of Temperate Grassland Species under Ambient and Elevated CO_2_: A Comparison of Fructan and Non-Fructan Accumulators

**DOI:** 10.1371/journal.pone.0092044

**Published:** 2014-03-26

**Authors:** Hamada AbdElgawad, Darin Peshev, Gaurav Zinta, Wim Van den Ende, Ivan A. Janssens, Han Asard

**Affiliations:** 1 Laboratory for Molecular Plant Physiology and Biotechnology, Department of Biology, University of Antwerp, Antwerp, Belgium; 2 Laboratory of Molecular Plant Biology, KU Leuven, Leuven, Belgium; 3 Research Group of Plant and Vegetation Ecology, Department of Biology, University of Antwerp (Campus Drie Eiken), Wilrijk, Belgium; University of Insubria, Italy

## Abstract

Elevated CO_2_ concentrations and extreme climate events, are two increasing components of the ongoing global climatic change factors, may alter plant chemical composition and thereby their economic and ecological characteristics, e.g. nutritional quality and decomposition rates. To investigate the impact of climate extremes on tissue quality, four temperate grassland species: the fructan accumulating grasses *Lolium perenne*, *Poa pratensis*, and the nitrogen (N) fixing legumes *Medicago lupulina* and *Lotus corniculatus* were subjected to water deficit at elevated temperature (+3°C), under ambient CO_2_ (392 ppm) and elevated CO_2_ (620 ppm). As a general observation, the effects of the climate extreme were larger and more ubiquitous in combination with elevated CO_2_. The imposed climate extreme increased non-structural carbohydrate and phenolics in all species, whereas it increased lignin in legumes and decreased tannins in grasses. However, there was no significant effect of climate extreme on structural carbohydrates, proteins, lipids and mineral contents and stoichiometric ratios. In combination with elevated CO_2_, climate extreme elicited larger increases in fructan and sucrose content in the grasses without affecting the total carbohydrate content, while it significantly increased total carbohydrates in legumes. The accumulation of carbohydrates in legumes was accompanied by higher activity of sucrose phosphate synthase, sucrose synthase and ADP-Glc pyrophosphorylase. In the legumes, elevated CO_2_ in combination with climate extreme reduced protein, phosphorus (P) and magnesium (Mg) contents and the total element:N ratio and it increased phenol, lignin, tannin, carbon (C), nitrogen (N) contents and C:N, C:P and N:P ratios. On the other hand, the tissue composition of the fructan accumulating grasses was not affected at this level, in line with recent views that fructans contribute to cellular homeostasis under stress. It is speculated that quality losses will be less prominent in grasses (fructan accumulators) than legumes under climate extreme and its combination with elevated CO_2_ conditions.

## Introduction

Global climate change conditions often alter plant chemical composition, which in turn can affect food and fodder quality, and decomposition rates [1]–[5]. These alterations in the chemical composition of plants grown under future climate will have significant impact on economical and ecological processes [1]–[5]. Therefore, it is pertinent to investigate the effects of climate change on plant chemical composition.

The anticipated climate changes are predominately associated with the rise in the concentrations of CO_2_ and a gradual rise in the earth's temperature, but also include increased frequency and intensity of extreme events (e.g. drought, heat wave and floodings) [6]. Effects of elevated CO_2_ in altering the chemical composition of plants have been extensively studied [7]–[12]. However, global climate change is characterized by the co-occurrence of co-varying environmental variables, which often affect plant chemical composition differently as when applied separately [4], [13], [14]. Up to our knowledge, studies illustrating the combined effect of elevated CO_2_ and climate extremes on the chemical composition of plants are scarce. An example of such a study is that of Larsen *et al*. [12] which indicated that single treatments of drought and elevated CO_2_ resulted in significantly increased C:N ratios, while ratios remained unchanged when drought and elevated CO_2_ were combined.

Drought stress and high temperature are important environmental factors, which restrict plant growth and alter tissue chemical composition [14], [15]. When applied separately, temperature [15] and drought [15] have been shown to alter the chemical composition of plants. Moreover, some studies have revealed that there is a significant interaction between drought and temperature in their effects on plant chemical composition [14], [12]. In this context, drought and elevated temperature altered the chemical composition by affecting carbohydrate concentrations. For example, under drought conditions, alfalfa accumulated starch, but lowered soluble sugars during vegetative growth [15]. Severe drought and elevated temperature significantly reduced the accumulation of free amino acids and soluble proteins in *Leymus chinensis* [14]. However, in future, higher temperatures and more frequent droughts will occur against a background of elevated CO_2,_ and very little is known about the combined effects of climate extremes under elevated CO_2_.

The anticipated changes in chemical composition are likely to alter tissue quality and decomposition rate. For example, elevated CO_2_ caused a reduction in forage quality through a lower crude protein content in different C_3_ and C_4_ plant species [16], and in combination with high temperature, reduced digestibility by enhancing fiber content in *Medicago sativa* [4]. Other CO_2_-induced tissue quality changes include an increased C/N ratio in soybean [17], and tannin accumulation in *Lotus corniculatus* [18]. The decreased tissue digestibility and decomposition rate associated with tannin accumulation is related to their ability to form digestion-resistant compounds with proteins as well as to direct inhibitory effects on microbial activity [5], [19], [20]. Altered stoichiometric ratios could affect the release of organic matter from decomposing materials by influencing the decomposers function [21]. On the other hand, elevated CO_2_ is likely to increase the tissue quality of fructan accumulating species via further increases in fructan and non-structural carbohydrate contents [22], [24]. In addition to their roles in plant stress tolerance, fructans are now widely recognized as fermentable food fibers and acknowledged for their health and immunomodulatory effects [25], [26]. Inulin-type fructans promote element resorption [27] and control insulin, cholesterol, triacylglycerol and phospholipid levels in animals and humans [28], [29]. Hence, to get a global overview on the tissue composition it is important to understand the metabolism under future climate conditions and to test the above mentioned metabolites to better assess the nutritional quality, digestibility and decomposability of plants. For this reason, we conducted a study in which we exposed plants to climate extreme under current and elevated CO_2_, and monitored the changes in a wide range of elements and metabolites.

Understanding changes in plant chemical composition in response to global change is further complicated because effects are species dependent. This is clear from the variation in response to elevated CO_2_ at the carbohydrate level (see above), but species responses also differ for other tissue quality parameters. Legumes had lower C:N, higher C:P, and higher N:P ratios than non-legumes when grown under elevated CO_2_ [30], related to their N fixing ability, more easily matching enhanced C assimilation [31], [32]. Barbehenn *et al*. [33] indicated that C_3_ grasses would have higher nutritional quality than C4 grasses under elevated [CO_2_], based on higher levels of proteins and fructans. Polyphenols were significantly increased in *Lolium perenne* compared to *Medicago lupulina* under climate extreme conditions [34]. It therefore appears worth to pay closer attention to compare climate effects on the chemical composition of different plant groups.

Grasslands cover 15% of the European land area and are important food sources for livestock [35], [36]. They are also an important component in the global C balance, by storing approximately one third of the terrestrial C stock. Changes in grassland growth and productivity resulting from changing climate conditions are therefore likely to have considerable impact on ecology and food resources. Thus, it is important to investigate the grassland species responses to climate changes.

Based on this knowledge, we here test the hypotheses that, 1) a climate extreme (water deficit under elevated temperature) affects the chemical composition of common temperate grassland species, which are important food sources for livestock, 2) that this effect is altered under elevated CO_2_, and 3) that these chemical changes differ among plant species and species groups. Specifically, we compared four grassland species, two fructan accumulating grasses and two non-fructan accumulating legumes.

## Materials and Methods

### Experimental set-up and plant harvest

#### Experimental set-up

A mesocosm experiment was conducted at the Drie Eiken Campus of Antwerp University, Belgium (51^o^ 09′ N, 04^o^ 24′ E, 10 m elevation). Seed of each species was sown in a non-climate controlled greenhouse and watered twice a week. After five weeks, seedlings of four temperate grassland species, two fructan accumulating grasses (*Lolium perenne* L., *Poa pratensis* L.) and two N-fixing legumes (*Medicago lupulina* L., *Lotus corniculatus* L.), were transplanted in 16 sunlit, south facing, climate controlled chambers (1). The interior surface area of each chamber was 1.5×1.5 m, height at the north side was 1.5 m and at the south side 1.2 m. The top of chambers consisted of a 4-mm thick colorless polycarbonate plate, whereas the sides were made of a 200-μm thick polyethylene film, both UV transparent. For each climate treatment four chambers were used and each chamber contained two populations (9 individuals with 5 cm interspace between them) of each species (Figure S1), grown in PVC tubes (19 cm diameter, 40 cm height) with sandy soil (96% sand, [37]. At the end, results of the two populations of each species, from the same chamber, were averaged yielding four biological replicates per climate treatment (i.e. n = 4). The climate scenarios ‘current’ and ‘future climate' were chosen according to the IPCC-SRES B2-scenario prediction of moderate change for the year 2100 [38]. In the past, numerous studies of CO_2_-only have already been conducted. Our experimental facilities included 16-growth chambers, of which only four chambers were equipped with CO_2_-control. Because the growth chamber was the unit of replication in this study, having a CO_2_-only treatment would have reduced the degree of replication to n = 2 or n = 3, with dramatic loss of statistical power. Instead of the typical orthogonal design, which would have allowed us to test the imposed of CO_2_-only, we therefore opted for an incremental design, focusing on the impact of drought stress: no drought, drought only, drought in a warmer climate, and drought in a warmer climate and higher CO_2_ climate. The detailed climate conditions in these four treatments were; 1) current climate, with ambient temperature and CO_2_ concentration (392±42 ppm) and sufficient water supply (Ambient, A); 2) drought stress in a current climate (D); 3) a climate extreme treatment, i.e. drought stress in a warmer climate (T_air_ +3°C); and, 4) a climate extreme (drought at elevated T) treatment combined with elevated CO_2_ (T_air_+3.02±0.82°C) (DTC); CO_2_ (615±81 ppm). Treatment D was omitted from this study, for two reasons. First, significant differences between D and DT conditions were very few. Second, the paper becomes unnecessarily complex because the significance levels of the difference between the D and A treatment were often different from those between the DT and A treatments.

The CO_2_ concentrations were monitored and maintained at the target concentration with a CO_2_ analyser (WMA-4, PP Systems, Hitchin, UK). The air temperature was monitored by a Siemens, type QFA66 sensor (Berlin, Germany). The temperature in current climate chambers followed the average daily air temperature course calculated for the period from 1996 to 2005. The temperature in the future climate treatments followed the same course but elevated by 3°C. Photosynthetic active radiation (PAR) was measured by a SDEC, type JYP1000 quantum sensor (SDEC, Reignac sur Indre, France). Microclimate parameters inside and outside each chamber were automatically logged every 30 min [39]. The average vapour pressure deficit was 0.35±0.02 and 0.46±0.02 kPa (SD) in the climate treatments with ambient and warmed air temperature, respectively. Irrigation was calculated from the monthly rainfall over the period 1995–2005 and corrected for differences in evapotranspiration (ET) inside and outside the chambers De Boeck *et al*. [40].

Drought was induced by withdrawing irrigation at 122 days after sowing for different periods of time. Plants were harvested for analysis when 50% of the drought-exposed population showed clear signs of stress: *i.e.* leaf discoloration, wilting and dehydration. This occurred after one week for *M. lupulina* and *L. corniculatus*, two weeks for *L. perenne* and three weeks for *P. pratensis*. These differences in the timing of stress occurrence mirrored declines of photosynthesis rate and stomatal conductance (see Figure S2). Harvesting of the above ground tissue of nine plants in each population was done by cutting plants 4 cm above the soil surface, rapid freezing in liquid nitrogen and storage at −80°C. For each species, the biochemical results of both populations per chamber were averaged yielding four replicates (chambers) per climate treatment.

### Metabolite Measurements

#### Carbohydrates

Small soluble sugars were determined in 0.2 g (FW) plant material, ground in liquid nitrogen (MagNALyser, Roche, Vilvoorde, Belgium) and extracted in 1 ml of 50 mM TAE buffer pH 7.5 (0.02% sodium azide, 10 mM mannitol, 0.1% polyclar, 10 mM NaHSO_3_, 1 mM mercapto-ethanol, 1 mM phenylmethanesulfonylfluoride (PMSF)). The extract was centrifuged (14,000 g, 4°C, 5 min), 150 μl was heated for 5 min in a water bath at 90°C. After cooling and centrifugation (14,000 g, 4°C, 5 min), the supernatant was added to a mixed bed Dowex column (300 μl Dowex H^+^, 300 μl Dowex Ac^–^; both 100–200 mesh; Acros Organics, Morris Plains, NJ, USA). The column was eluted six times with 150 μl of ddH_2_O. Glucose, fructose, sucrose and raffinose concentrations were measured by HPAEC-PAD as before [41]. In legumes (*M. lupulina* and *L. corniculatus*), total soluble sugars was calculated as a sum of the measured individual soluble sugars, whereas total fructan was also added in the case of grasses (*L. perenne* and *P. pratensis*). Total fructan levels and fructan patterns were generated as described [42].

The remaining pellet of soluble sugars extraction was treated with a mixture of α-amylase and amyloglucosidase to extract starch (100 U/ml, 1 h, 45°C, [43]. Sugar concentrations in the total soluble and starch extracts was estimated by the anthrone reagent method. Cellulose was extracted from 0.2 g DW plant material, by boiling in 100°C nitric acid/acetic acid (1∶8, v/v, 1 h) to remove lignin, hemicellulose and xylosans after successive centrifugations at 14,000 for 15 min, and dilution with 67% H_2_SO_4_ (v/v). Cellulose was determined at 620 nm using the anthrone reagent.

#### Soluble and total protein

Frozen plant material (0.2 g FW) was homogenized by MagNALyser in 2 ml of cold 0.05 M K-phosphate buffer (pH 7.0) and centrifuged (14,000 g, 4°C, 20 min). The supernatant was treated with 10% (w/v) TCA to precipitate soluble protein, which were redissolved in 1 N NaOH. The remaining pellet was used to measure insoluble proteins. It was successively washed with 80% ethanol (v/v), 10% (w/v) cold TCA, ethanol:chloroform (3∶1, v/v), ethanol:ether (3∶1, v/v), and ether to remove phenolic compounds. The washed pellet was re-dissolved in 1 N NaOH at 80°C for 1 h and soluble and insoluble protein content was estimated [44]. Total protein content was calculated by adding the contents of soluble and insoluble proteins.

#### Lignin, polyphenols and tannin

For lignin determination, 0.1 g DW plant material was homogenized (MagNALyser) with 2 ml 95% ethanol and centrifuged (14,000 g, 4°C, 3 min). The pellet was washed with different organic solvents at high temperatures, then 1 ml of 25% acetyl bromide in acetic acid (1∶3, v/v) was added to the pellet and incubated at 70°C for 30 min. After cooling, 0.2 ml of 2 M NaOH and 0.1 ml of 7.5 M hydroxylamine hydrochloride were added, and the volume was made up to 10 ml with acetic acid. After centrifugation (1,000 g, 5 min), the absorbance was measured at 280 nm [45]. Polyphenols were extracted in 2 ml 80% ethanol (v/v) (0.2 g DW, MagNALyser) and determined with gallic acid as the standard [46]. Tannin content was determined [47] by homogenizing (MagNALyser) 0.2 g FW tissue in 2 ml 0.1 M acetate buffer pH 5, containing 2 mg of bovine serum albumin, incubated for 15 min at room temperature and centrifuged (14,000 g, 4°C, 15 min). The pellet was dissolved in 4 ml a 1% (w/v) SDS and 5% (v/v) tri-ethanolamine solution. One ml of 10 mM FeCl_3_ in 0.01 N HCl was added and the absorbance determined at 510 nm. Tannic acid was used as the standard.

#### C, N, macro-minerals and trace elements

C and N contents were measured with a CN element analyser (NC-2100, Carlo Erba Instruments, Milan, Italy). For Macro-minerals and trace elements, 100 mg DW plant material was digested in a 5∶1 ratio of HNO_3_/H_2_O in a microwave oven and determined by mass spectrometry (ICP-MS, Finnigan Element XR, Scientific, Bremen, Germany). A mixture of standards was prepared in 1% nitric acid.

### Enzyme activity measurements

#### Carbohydrate metabolism enzymes

Neutral and soluble acid invertases were extracted in five volumes of the same ice-cold TAE extraction buffer (pH 7.5) as used for the small soluble sugar analysis (see above) and then centrifuged at 14,000 g, 15 min at 4°C. The pellet was washed three times with ice-cold 50 mM Na-acetate buffer, pH 5.0 and redissolved in this buffer. Aliquots of this suspension were subsequently used for cell wall activity determinations under continuous shaking at 30°C (500 rpm; Thermomixer®, eppendorf) to keep the walls in suspension. The supernatant was split in two parts and used for the determinations of soluble acid and neutral invertase activities, respectively. After precipitation by 80% saturated (NH_4_)_2_SO_4_, (incubation on ice for 30 min, centrifugation at 14 000 g, 4°C, 5 min), the pellets were washed three times with 800 μl of 80% (NH_4_)_2_SO_4_-saturated in TAE buffer pH 8.5 (neutral invertases) and Na-acetate buffer, pH 5.0 (soluble acid invertases). Finally, pellets were dissolved in 150 μl 50 mM Na-acetate buffer, pH 5.0 (soluble acid invertase and in TAE buffer pH 8.5 (neutral invertases). Invertase activity was determined in 100 μl reaction mixtures containing 100 mM sucrose in TAE buffer pH 8.5 or Na-acetate buffer pH 5.0 containing 0.02% (w/v) Na-azide. Reaction mixtures were incubated at 30°C, and the reactions were stopped by keeping an aliquot for 5 min in a water bath at 90°C. Fructose concentrations were measured as described above.

Sucrose phosphate synthase and sucrose synthase were extracted (MagNALyser) from 0.2 g fresh plant material in 1 ml HEPES buffer (100 mM HEPES pH 8.2, 10 mM EDTA, 15 mM KCl, 5 mM MgCl_2_, 2 mM sodium diethyl dithiocarbamate, 5 mM β-mercaptoethanol, 1% PPV). After centrifugation (14,000 g, 4°C, 15 min) the supernatant was cleaned [48]. Sucrose synthase activity was measured [49] by measuring the reduction of NAD^+^ at 340 nm, in a reaction mixture containing 100 mM bicine KOH buffer (pH 8.5, 100 mM sucrose, 2 mM UDP, 0.025 U UDP-glucose dehydrogenase, 1.5 mM NAD^+^). Sucrose phosphate synthase was measured in 1 ml of HEPES buffer (20 mM HEPES, pH 8.2, 2.2 mM UDP- glucose, 4.4 mM fructose-6-phosphate, 1 mM MgCl_2_, 2 mM NaF) at 37°C for 15 min, and stopped by adding 30% NaOH and 10 min boiling. Sucrose concentrations were determined with the anthrone reagent, as described above.

ADP-Glc pyrophosphorylase was extracted (MagNALyser) from 0.2 g FW plant samples [50]. After centrifugation (14,000 g, 4°C, 5 min), the supernatant was mixed with 80 mM HEPES buffer (pH 7.4, 10 mM MgCl_2_, 1 mM ADP-glucose, 0.6 mM NAD^+^, 10 μM glucose-1,6- phosphate, 2,3 mM DTT, 0.02% BSA, 1 U phosphoglucomutase, 2.5 U NAD-linked glucose-6-phosphate dehydrogenase), and the activity measured as the NAD^+^ reduction at 340 nm and 30°C.

#### Phenol and lignin biosynthesis enzymes

PAL was extracted from 0.2 g (FW) frozen plant material in 1 ml sodium borate buffer (200 mM, pH 8.8, Koukol and Conn, 1961), and assayed by measuring the absorbance of trans-cinnamic acid at 290 nm. For the cinnamyl alcohol dehydrogenase activity analysis, 5 g (FW) tissue was extracted in 10 ml Tris:HCl buffer (200 mM Tris, pH 7.5), the activity was measured by monitoring the production of cinnamyl aldehyde at 400 nm [51].

### Statistical analysis

The data were analyzed by procedure of the Statistical Analysis System (SPSS Inc., Chicago, IL, USA). The assumptions of normality of distribution and homogeneity of variance were examined. Since both assumptions were met, transformations were not necessary and analysis of variance (ANOVA) was done on the original data. The impact of climate treatments: no drought, drought in a warmer climate, and drought in a warmer climate and higher CO_2_ climate was tested by one-way ANOVA procedure. Number of replicates (chambers) for each climate treatment was four (n = 4). The significant differences between the means were determined by using the Duncan test (P<0.05). Multiple testing corrections were carried out by using Benjamini and Hochberg false discovery rate (FDR). The relationships between treatment variables were analyzed by using Pearson correlation.

## Results

### Carbohydrate metabolism

**Figure 1:**
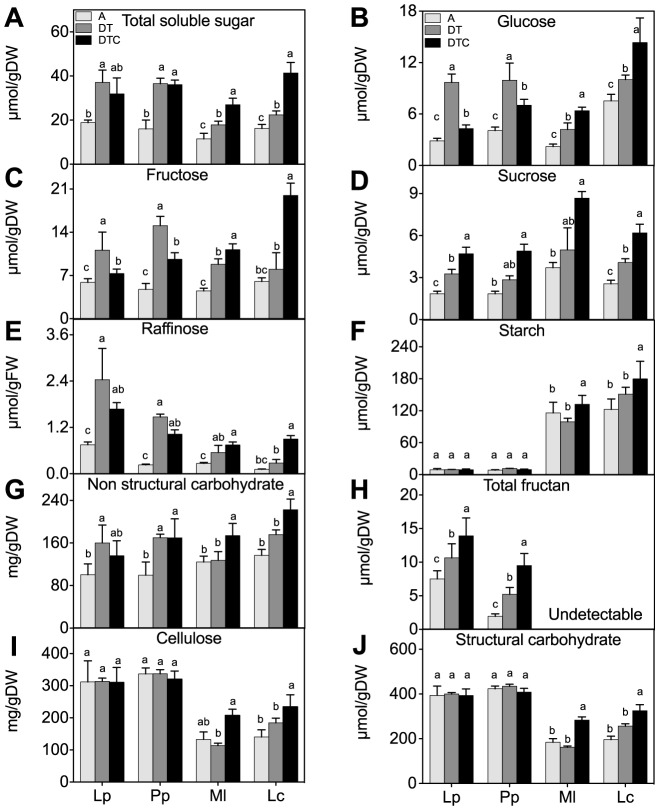
Effect of climate extreme conditions in ambient and elevated CO_2_ on carbohydrate fractions. Climate conditions: ambient (A), drought and warming (DT), drought and warming in elevated CO_2._ Grassland species: *Lolium perenne* (Lp), *Poa pratensis* (Pp), *Medicago lupulina* (Ml) and *Lotus corniculatus* (Lc). The different panels show, Total soluble sugar (A), Glucose (B), Fructose (C), Sucrose (D), Raffinose (E), Total fructan (F), Non structural carbohydrate (G), Starch (H), Cellulose (I) and Structural carbohydrate (J). Different letters in the graph represent significant differences between A, DT and DTC at p<0.05 (n = 4).

**Figure 2:**
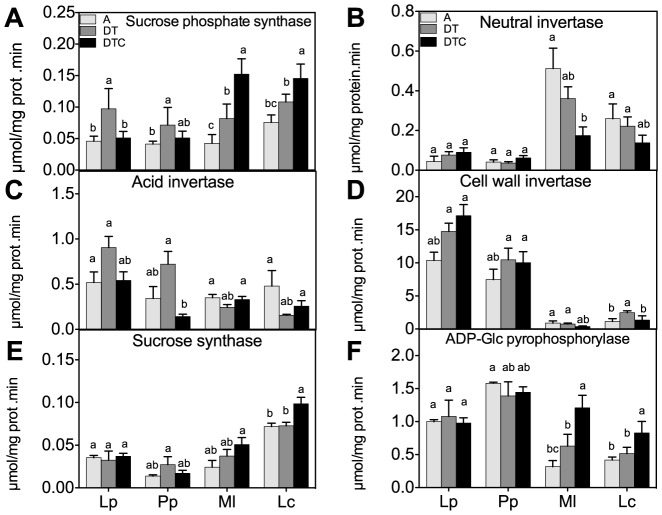
Effect of climate extreme conditions in ambient and elevated CO_2_ on carbohydrate metabolism enzyme activities. Climate conditions: ambient (A), drought and warming (DT), drought and warming in elevated CO_2._ Grassland species: *Lolium perenne* (Lp), *Poa pratensis* (Pp), *Medicago lupulina* (Ml) and *Lotus corniculatus* (Lc). The different panels show Sucrose phosphate synthase (A), Neutral invertase (B) Soluble acid invertase (C), Cell wall invertase (D), Sucrose synthase (E) and ADPG pyrophosphorylase (F). Different letters in the graph represent significant differences between A, DT and DTC at p<0.05 (n = 4).

The difference in fructan and starch accumulation between the grasses and legumes is clear (Figure 1E and H), as well as the strongly differing cell wall and neutral invertase activities (Figure 2B and D). In all four species, the total soluble sugar and non-structural carbohydrates content significantly increased under climate extreme conditions (DT) (Figure 1A and G). This increase was also often reflected in the contents of individual soluble sugars, glucose, fructose, sucrose, raffinose and total fructan (Figure1 B, C, D, E and F). As compared to the two legumes, fructans were detected only in the two grasses and were further accumulated by climate extreme conditions. There was generally no effect of the climate extreme on the accumulation of starch (Figure 1H), cellulose (Figure 1I) and total structural carbohydrates (Figure 1J).

Elevated CO_2_ (DTC) magnified the climate-mediated induction of the sucrose and fructan contents and counteracted the increase of hexoses in the grasses, without significantly changing the total soluble sugar content. On the other hand, elevated [CO_2_] further increased all carbohydrates levels in the two legumes (Figure 1). A species-specific pattern was also observed at the level of the total structural and non-structural carbohydrate content, and at the starch and cellulose levels; i.e. as compared to climate extreme, elevated CO_2_ did not affect, or even slightly decreased, these carbohydrates in *L. perenne* and *P. pratensis*, but significantly increased their levels in *M. lupulina* and *L. corniculatus*.

Sucrose phosphate synthase, different types of invertases, sucrose synthase and ADP-Glc pyrophosphorylase are key enzymes in sucrose and starch metabolism. Sucrose phosphate synthase mediates the synthesis of sucrose-6-phosphate [52], whereas sucrose synthase and invertases are involved in sucrose degradation [53], affecting subsequent starch synthesis via ADP-Glc pyrophosphorylase [54]. The climate extreme conditions had different effects on these enzymes. Sucrose phosphate synthase activity (Figure 2A) was induced by drought and elevated temperature (DT) in all species. However, these conditions had little effect on invertase activity, i.e. neutral-, acid soluble invertase and acid cell wall-invertases (Figure 2B, C and D respectively) in either species. Similarly, no clear changes in sucrose synthase and ADP-Glc pyrophosphorylase activity were observed in either species under the climate extreme (Figure 2E, F).

As was observed at the level of the soluble sugar concentrations, elevated [CO_2_] reduced the effect of the climate extreme on sucrose phosphate synthase in the grasses. However, the opposite was observed in the legumes where elevated CO_2_ resulted in increased soluble sugar levels and sucrose phosphate synthase activities. At the invertase level, increased CO_2_ did not alter the impact of the climate extreme, except in the legumes, where the activity of the neutral invertase and cell wall invertases strongly decreased as compared to the DT condition. By contrast, sucrose synthase and ADP-Glc pyrophosphorylase activities were induced in the legumes by 36 and 34% and by 92 and 60% in *M. lupulina* and *L. corniculatus* respectively, under the combined treatment of climate extreme and elevated CO_2_.

### Constituents affecting plant nutritional quality and decomposition rate

**Figure 3:**
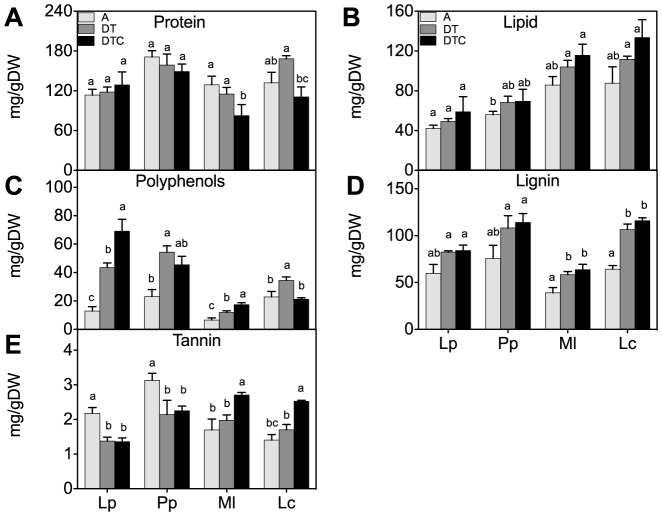
Effect of climate extreme conditions in ambient and elevated CO_2_ on metabolite classes. Climate conditions: ambient (A), drought and warming (DT), drought and warming in elevated CO_2._ Grassland species: *Lolium perenne* (Lp), *Poa pratensis* (Pp), *Medicago lupulina* (Ml) and *Lotus corniculatus* (Lc). The different panels show Protein (A), Lipid (B), Lignin (C), Polyphenols (D) and Tannins (E). Different letters in the graph represent significant differences between A, DT and DTC at p<0.05 (n = 4).

The climate extreme generally had no or little effect on the total protein and lipid contents (Figure 3A and B). The treatment significantly increased the polyphenol content in all species except *L. corniculatus* (Figure 3C). Lignin, an indigestible polymer, showed no significant changes under the climate conditions in both grass species, whereas higher lignin contents were observed in the legumes (Figure 3D). Tannins are commonly found metabolites that possess anti-nutritional and anti-feed properties [5]. Tannins did not change in both legume species, but markedly decreased in the two grasses as a result of the drought and warming (44 and 39%, respectively) (Figure 3E).

Increasing the CO_2_ concentration affected the response to drought and warming of some of measured tissue quality parameters. Elevated CO_2_ did not alter the lipid or lignin content in any of the species, but it significantly reduced the total protein content in the legumes (Figure 3A). Similarly, elevated CO_2_ did not alter the climate extreme-response of the polyphenols and tannins in the grasses, while it significantly increased the tannins in the legumes.

**Figure 4:**
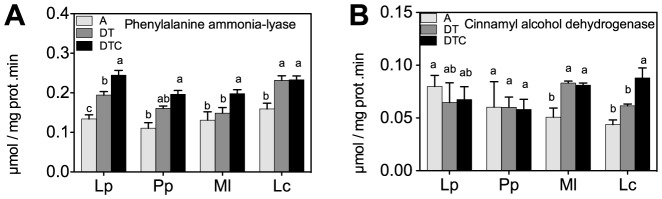
Effect of climate extreme conditions in ambient and elevated CO_2_ on polyphenol metabolism enzyme activities. Climate conditions: ambient (A), drought and warming (DT), drought and warming in elevated CO_2._ Grassland species: *Lolium perenne* (Lp), *Poa pratensis* (Pp), *Medicago lupulina* (Ml) and *Lotus corniculatus* (Lc). The different panels show Phenylalanine ammonia-lyase (A) and Cinnamyl alcohol dehydrogenase (B). Different letters in the graph represent significant differences between A, DT and DTC at p<0.05 (n = 4).

To better understand the mechanism underlying the changes in polyphenol and lignins, we measured phenylalanine ammonia lyase and cinnamyl alcohol dehydrogenase, the key enzymes in their respective biosynthetic pathways. Phenylalanine ammonia lyase activity increased slightly by the climate extreme treatment in most species, and strongest in *L. corniculatus* (Figure 4A). Activity of cinnamyl alcohol dehydrogenase increased only in the legumes (Figure 4B). Elevation of CO_2_ levels in the drought and warming treatment (DTC) generally did not significantly alter phenylalanine ammonia lyase and cinnamyl alcohol dehydrogenase activities, with the exception of an increase in the activity of cinnamyl alcohol dehydrogenase in *L. corniculatus*.

**Figure 5:**
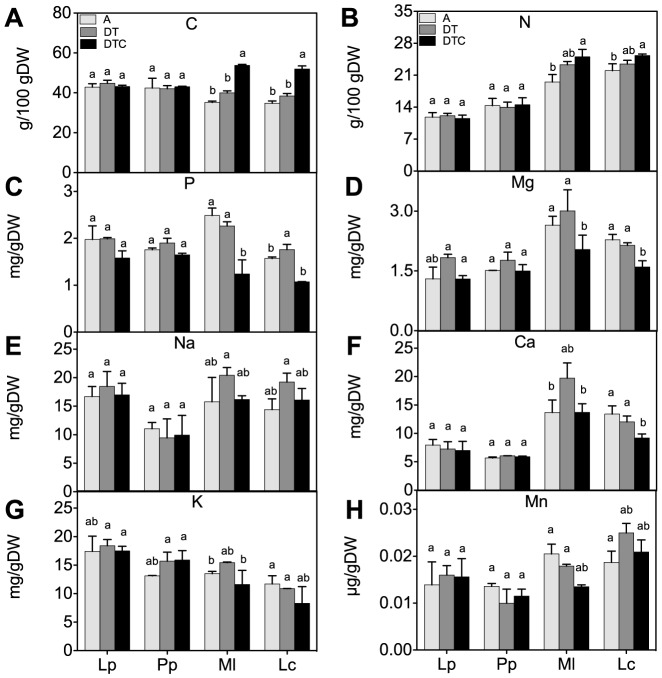
Effect of climate extreme conditions in ambient and elevated CO_2_ on macronutrients and trace elements. Climate conditions: ambient (A), drought and warming (DT), drought and warming in elevated CO_2._ Grassland species: *Lolium perenne* (Lp), *Poa pratensis* (Pp), *Medicago lupulina* (Ml) and *Lotus corniculatus* (Lc). The different panels show C (A), N (B), P (C), Mg (D), Na (E), Ca (F), K (G) and Mn (H). Different letters in the graph represent significant differences between A, DT and DTC at p<0.05.

**Figure 6:**
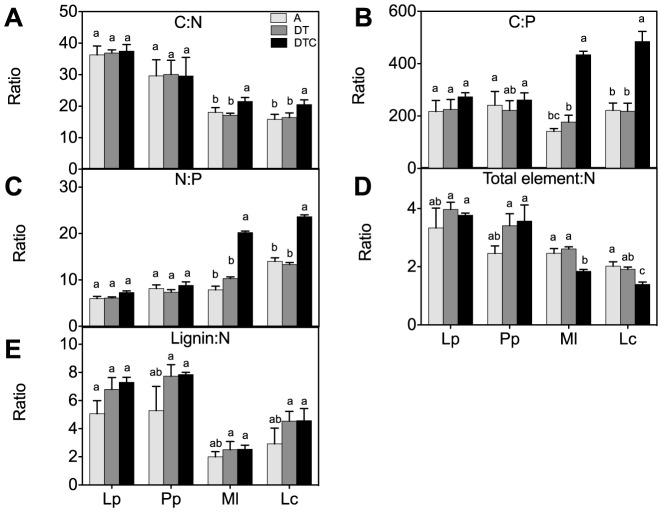
Effect of climate extreme conditions in ambient and elevated CO_2_ on stoichiometric ratios. Climate conditions: ambient (A), drought and warming (DT), drought and warming in elevated CO_2._ Grassland species: *Lolium perenne* (Lp), *Poa pratensis* (Pp), *Medicago lupulina* (Ml) and *Lotus corniculatus* (Lc). The different panels show C:N ratio (A), C:P ratio (B), N:P ratio (C), Element: P ratio (D) and Lignin: N ratio (E). Different letters in the graph represent significant differences between A, DT and DTC at p<0.05 (n = 4).

Net rates of nutrient release during plant decomposition are strongly related to their initial concentration and stoichiometry [55]. We found that there was no significant effect of the climate extreme on the concentrations of macronutrients (N, C, P, Ca, Mg, Na and K) or trace elements (Cu and Mn) in all four-plant species (Figure 5 A, B, C, D, E and F). The climate extreme also did not significantly affect aboveground plant stoichiometry in either species (Figure 6).

Elevated CO_2_ in combination with climate extreme (DTC) increased the content of C and N and decreased the concentrations of P and Mg in the legumes (Figure 5 A, B, C and D), relative to those in the extreme only (DT) treatment. Importantly, these decreases in P and Mg concentrations were not significant in the grasses. Elevated CO_2_ only affected element ratios in the legumes, but not in the grasses. The legumes showed significant increases in C:N and C:P ratios through the increased C concentration (Figure. 6A, B). The increased N:P ratio in legumes may in part be explained by increases in N content, but also results from a decreased P content (Figure. 5A). Also the total element:N ratio was reduced significantly by elevated CO_2_ in the two legumes (Figure 6D).

### Are changes in enzyme activities and metabolites correlated?

To unravel the importance of the enzymes in controlling the respective carbohydrate metabolite levels, a correlation analysis was performed. There was a statistically significant, positive correlation between the change in sucrose phosphate synthase activity and the change in the sucrose content (Table 1, r = 0.872, p<0.05). We also found a positive correlation between sucrose synthase activity and starch content (Table 1, r = 0.549, p<0.05). Oppositely, there was no correlation between the invertases activities and the change in sucrose content. There was also a positive correlation between ADP-Glc pyrophosphorylase activity and starch levels (Table 1, r = 0.739, p<0.05). Consistent with the absence of variation in the starch contents in both grasses, no effects of climate extreme and elevated CO_2_ conditions on ADP-Glc pyrophosphorylase activity was observed.

**Table 1:** Correlations among various variables of four studied grassland species.

Parameters	Cellulose	SUS	Sucrose	SPS	NI	AI	CWI	Starch	AGPASE	Total SS	Fructan	Protein	Lipid	Phenols	PAL	Lignin	CAD
SUS	0.874**																
Sucrose	0.072	0.260															
SPS	1.000**	0.874**	0.872**														
NI	−0.068	−0.014	−0.070	−0.068													
AI	0.018	−0.095	−0.299	0.018	−0.417												
CWI	−0.107	−0.257	−0.334	−0.107	−0.699**	0.668**											
Starch	0.122	0.549*	0.781**	0.122	0.143	−0.364	−0.559*										
AGPASE	0.259	−0.053	0.024	0.259	−0.706**	0.194	0.471	0.739**									
Total SS	0.004	−0.115	−0.026	0.004	−0.913**	0.473	0.802**	−0.304	0.740**								
Fructan	0.167	0.430	0.891**	0.167	0.096	−0.319	−0.531*	0.958**	−0.243	−0.245							
Protein	0.487	0.182	−0.307	0.487	−0.155	−0.015	0.159	−0.368	0.537*	0.170	−0.528						
Lipid	0.099	0.286	0.654**	0.099	0.500*	−0.611*	−0.802**	0.794**	−0.414	−0.689**	0.798**	−0.188					
Phenols	0.021	−0.032	0.059	0.021	−0.626	0.402	0.713**	−0.140	0.415	−0.085	0.609*	0.072	−0.324				
PAL	−0.134	0.079	0.564*	−0.134	−0.210	−0.080	0.153	0.541*	−0.155	0.074	0.559*	−0.177	0.390	0.525*			
Lignin	0.078	0.166	0.641**	0.078	−0.578	−0.055	0.267	0.413	0.367	0.455	0.466	−0.061	0.172	0.638**	0.620*		
CAD	0.236	0.223	0.326	0.236	−0.089	−0.062	0.022	0.079	0.142	0.100	0.202	0.099	0.072	−0.270	0.075	0.347	
Tannin	0.180	−0.079	−0.385	0.180	−0.283	−0.072	0.176	−0.526*	0.551*	0.325	−0.554*	0.205	−0.429	0.032	−0.645**	0.023	−0.207

**Correlation is significant at P<0.01 (2-tailed).

*Correlation is significant at P<0.05 level (2-tailed).

SUS =  Sucrose synthase, SPS =  Sucrose phosphate synthase, NI =  Neutral invertase, AI =  Acid invertase, CWI =  Cell wall invertase, AGPASE =  ADP-Glu pyrophosphorylase, Total SS =  Total soluble sugar, PAL =  Phenylalanine ammonialyase, CAD =  Cinnamyl alcohol dehydrogenase.

At the level of other metabolites and enzymes, we found a significant correlation between phenylalanine ammonia lyase activity and the phenolic content (Table 1, r = 0.525, p<0.05). However, despite the observation that increases in the lignin content in the two legume species were accompanied by higher cinnamyl alcohol dehydrogenase activity in climate extreme conditions, there was no strong correlation between lignin content and the enzyme activity (Table 1, r = 0.347, p<0.05).

We also observed correlations between changes in metabolite levels. There was a negative correlation (Table 1, r = −0.528, p<0.05) across all species, between the non-structural carbohydrate and the protein content. This was particularly apparent in the legumes and suggests C being reallocated from proteins to sugars. Also, there was a strong correlation between accumulation of sucrose, soluble sugar and non-structural carbohydrate and the accumulation of polyphenols and tannins (Table 1, r = 0.822, 0.705 and 0.609 respectively, p<0.05).

## Discussion

### Climate extreme conditions affect plant chemical composition

To evaluate changes in grassland food properties, we analysed the carbohydrate, lipid, protein and element composition of four grassland species (*L. perenne*, *P. pratensis, M. lupulina* and *L. corniculatus*) subjected to a climate extreme, water deficit combined with elevated temperature (+3°C), under ambient and elevated CO_2_. In climate extreme conditions all plants accumulated more soluble sugars and polyphenols. In addition, the legumes also had increased lignin content and the grasses showed decreased tannin levels. These results are consistent with previous observations, such as increases in soluble sugars in *Phaseolus vulgaris* leaves under temperature stress [56], and increases in tannin, polyphenols and lignin as a result of drought and/or high temperature stress in Lotus, *Lolium* and *Medicago* [15], [34], [57], [58]. A reduction in tannin contents was also observed under drought stress in *L. corniculatus* leaves [18].

The climate extreme conditions did not affect insoluble sugars, proteins, lipids and minerals, or plant (element) stoichiometric ratios. Similarly, little or no significant changes in total protein, mineral or starch were reported under drought stress in *Lolium perenne* and Tobacco plant [59], [60]. This is in contrast to results reported by others, in which water deficit stress induced changes in these parameters. For example, high temperature and drought, or their combination, increased starch content in *M. sativa* [15], and decreased protein and lipid contents in *Leymus chinensis* and soybean [1], [14]. The variations in responses at the plant composition level may be attributed to variation in the magnitude of the stress conditions, as well as to different plant species.

The accumulation of soluble sugars and polyphenols, in adverse climate conditions, are probably examples of adaptive protection strategies [61], [62]. Polyphenols constitute a large group of diverse molecules, implemented in ROS detoxification and in the protection of the photosynthetic apparatus [63], [64]. Higher amounts of soluble sugars provide more substrate for other defense responses [65]. However, these changes in chemical composition not only enhance plant protection, they will likely also affect the nutritional value and digestibility of these species [2], [3]. Accumulation of polyphenols (toxic compounds) and lignin (indigestible fiber) decreased plant quality in economic point of view [66], [67], [68]. Polyphenols can also retard microbial and enzymatic decomposition by forming resistant compounds or by inhibiting microbial activity [20], [69]. The stress-induced increase in polyphenols is probably explained by parallel increases in phenylalanine ammonia lyase activity, a key enzyme in polyphenol biosynthesis [61] (e.g. Table 1).

### Elevated CO_2_ alters the climate extreme impact

With the continued rise in atmospheric CO_2_, the effect of climate extremes is best evaluated against an elevated CO_2_ background. In *L. perenne, P. pratensis, M. lupulina* and *L. corniculatus*, elevated CO_2_ in climate extreme conditions enhanced several aspects of the carbohydrate and secondary metabolite metabolism. In the legumes, significant increases in the soluble sugar, starch, cellulose, structural carbohydrate, polyphenol and tannin contents were observed. On the other hand, in the grasses, combination of elevated CO_2_ with climate extremes increased sucrose and fructan contents and reduced hexoses.

Increases in structural and non-structural carbohydrates due to CO_2_ enrichment have been reported, and may be explained by higher carbohydrate assimilation rates [70], [71], [72]. The carbohydrate accumulation in legumes was accompanied by increased activities of sucrose phosphate synthase and ADP-Glc pyrophosphorylase (Figure 2) as was also previously observed in *Phaseolus* leaves [56]. Interestingly, it appears a switch occurred in cytosolic sucrose catabolism from neutral invertase to sucrose synthase in the legumes, but not in the grasses (Figure. 2). Increased sucrose synthase activities are also linked to increased starch synthesis, although the underlying mechanisms require further exploration [73], [74]. *L. perenne* and *P. pratensis* are fructan-accumulating grasses, and elevated CO_2_ has previously been observed to allocate to fructans in fructan accumulators [24], [75].

At the level of secondary metabolites, there is a notable increase in tannin and lignin content, under stress in elevated CO_2_ conditions in the legumes. Similar observations have been attributed to increased shikimate metabolism in high C availability [76], [77]. The accumulation of tannins in legumes can retard decomposition [20], [23], [68].

Frequently observed changes in tissue chemistry, induced by changing climate conditions *e.g*., elevated CO_2_ and/or drought and temperature, include decreases in protein content [7], [8], [14]. In our experiment, elevated CO_2_ in climate extreme reduced protein level mostly in the legumes (Figure. 3). A decrease in tissue protein content lowers its nutritive quality [68], [78]. Given the increases in carbohydrates, it appears that the part of the tissue C is reallocated from proteins to carbohydrates under elevated CO_2_ in the legumes. Importantly, fructan-accumulating grasses did not show such decreased protein levels under elevated CO_2_. It can be speculated that these species keep their C fluxes towards fructans and polyphenols (as stress tolerance contributors) and proteins (economic importance), while they are diverting none (or less) extra C to cellulose, other structural carbohydrates, lignins and tannins.

Overall, strong correlations were observed between accumulation of sucrose, total soluble sugar and non-structural carbohydrate and the accumulation of polyphenols. It is well-known that sucrose specific signalling mechanisms trigger polyphenol synthesis pathways [26], [79]. In Arabidopsis, soluble sugars, anthocyanins and proline typically increase together under water-deficit stress [79], and in chicory, exogenous sucrose feeding leads to the combined increase in polyphenols and fructans [80]. In addition to their direct ROS scavenging effects, fructans are recently proposed as (secondary) stress signals stimulating innate immunity responses, in plants and animals [26], [80]. Both fructans and polyphenols, such as anthocyanins, might play roles both in abiotic and biotic stress responses [81].

Increasing CO_2_ under climate extreme did not affect the element composition in the grasses, but several elements, most prominently C and N increased, and P and Mg decreased, in the legumes (Figure. 5). In general, reductions in P and Mg in legumes could lower their food quality for herbivores [82], [83]. As a result of the changes in P, also the C:P and N:P ratios change considerably in the legumes. Elevated CO_2_ has often been observed to decrease nutrient concentrations of plant tissues [84]. These stress and climate-induced stoichiometry changes are therefore likely to affect food nutritional quality and decomposition [82], [83], [85], [21].

Under combination of elevated CO_2_ concentration with drought or temperature, plants typically show increased tissue C concentrations [24], [86], with correspondingly reduced concentrations of other elements, including N [87], and several trace elements [85]. In agreement [30], we also found that elevated CO_2_ in climate extreme increased C:P and N:P ratios in legumes only. The increase in C:P ratio (e.g. Figure 6B, is not only explained by elevated C accumulation, but also by a lower P content. The lignin:N ratio is another factor that could affect decomposition rates [88], but this parameter did not change considerably in extreme climate conditions.

### Climate extreme and elevated CO_2_ effects are species-group specific

Plant responses to climate change is complicated by significant ‘species x climate’ interactions and show species-group specific responses. For example, plant functional types differed in their stoichiometric ratios under elevated CO_2_ [30]. Also, fructan accumulating grasses showed higher tissue quality than non-fructan accumulators and showed increase in proteins and fructans levels under elevated CO_2_ [33]. Accumulation of fructan content in species, improve their protein utilization by livestock [89] and digestibility by ruminants [29], [33]. For this reason we estimated responses of different species to climate change conditions.

Grasses and legumes responded differently to extreme climate conditions and elevated CO_2_ in various tissue composition parameters. At the level of carbohydrate metabolism, soluble sugar levels were stress-induced in all species. However, fructan levels were only increased in the grasses. Moreover, elevated CO_2_ also affected sugar metabolism differentially between legumes and grasses, with a noteworthy shift in sugar metabolism in the legumes, where non-structural and structural carbohydrates were increased. At least parts of these differences relate to the N-fixing potential, supporting more and higher C sinks [90]. Consistently, interactive effects of elevated CO_2_ and plant species on starch and sugar concentrations were observed in previous study [91].

Also other tissue composition parameters were affected differently between these species groups, in particular at elevated CO_2_. For example, in contrast to the grasses, protein, P and Mg levels decreased, and polyphenol, tannin and lignin levels increased in the legumes under climate extreme and elevated CO_2_. Moreover, in legumes element's stoichiometry were affected (C:N, C:P, N:P and element:N). As legume are N-fixing species, high N and low P content resulted in a high N:P ratio compared to grasses. This increase was also observed in previous studies [30], [91].

Together, these findings suggest that future climate elevated CO_2_, combined with climate extreme conditions, may reduce tissue digestibility and quality of legume species. It also appears that the fructan accumulating grasses preserved their tissue quality (protein, macro and micronutrients) better under stress and elevated CO_2_, partially at the expense of the deposition of cell wall materials. This observation fits with reports that fructan accumulating plants such as chicory behave well under stress [92], but show a growth lag phase possibly related to slower deposition of new cell wall materials [93], [94]. It can be speculated that fructan accumulators invest more in defense responses during early developmental stages, even in the absence of stress. This is consistent with the strongly increased cell wall invertase activities in fructan accumulators (Figure 2D). Increased cell wall invertase activities are typically associated with stress responses [95] and tolerant genotypes maintain higher cell wall invertase activities, even in the absence of stress [96]. Cell wall invertases are emerging as important regulators of apoplastic sugar homeostasis [97] associated with altered sugar signaling events and C partitioning [79], [26], [96].

## Conclusions and perspectives

The effect of climate extreme treatment (water deficit at elevated background temperature) was more pronounced at elevated CO_2_, in particular in legumes. Interactions between elevated CO_2_ and climate extremes were observed in many cases, where elevated CO_2_ amplified or reduced the impact of the climate extreme. The results also support the importance of the variation in responses among species groups. Growth of legumes under extreme climate and elevated CO_2_ conditions resulted in large compositional changes, while minor changes in tissue chemistry of the fructan-accumulating grasses were observed. This suggests that quality losses may be more prominent in non-fructan accumulators. Further research is needed to the roles of fructans as antioxidants and putative (stress) signals affecting C partitioning. For this purpose, metabolite pools in fructan-accumulating transgenic crops and wild-type crops should be rigorously compared under stress and/or elevated CO_2_ conditions.

## Supporting Information

Figure S1 **Experimental design.** Design of the 16 climate-controlled chambers (A) and internal arrangement of pots in chamber 1 (B).
(EPS)

Figure S2 **Effect of climate extreme on photosynthesis and stomatal conductance.** Climate conditions: ambient (A), drought and warming (DT), drought and warming in elevated CO_2_] Grassland species: *Lolium perenne* (Lp), *Poa pratensis* (Pp), *Medicago lupulina* (Ml) and *Lotus corniculatus* (Lc). The different panels show photosynthesis (A) and stomatal conductance (B) after 0,1,2 and 3 weeks of drought stress.
(EPS)
